# Peripapillary microvasculature changes after vitrectomy in epiretinal membrane via swept-source OCT angiography

**DOI:** 10.1186/s12886-023-02793-9

**Published:** 2023-02-06

**Authors:** Kyungwoo Yoon, Jong Beom Park, Min Seok Kang, Eung Suk Kim, Seung-Young Yu, Kiyoung Kim

**Affiliations:** Department of Ophthalmology, Kyung Hee University Hospital, Kyung Hee University, Seoul, Korea

**Keywords:** Swept-Source Optical Coherence Tomography Angiography (SS-OCTA), Epiretinal membrane, Vitrectomy, Peripapillary capillary density

## Abstract

**Purpose:**

To evaluate the peripapillary microvasculature changes in patients with epiretinal membrane (ERM) following pars plana vitrectomy (PPV) with internal limiting membrane (ILM) peeling using swept-source optical coherence tomography angiography (SS-OCTA).

**Method:**

Medical records and multimodal imaging data of 33 eyes after PPV for ERM were retrospectively reviewed. Peripapillary SS-OCTA images of 6×6 mm^2^ were recorded at at pre- and post-operatively every 6 months for 1 year. A semi-automated method was used to analyzed SS-OCTA images, excluding the optic disc area, using the MATLAB software. The peripapillary vessel density (pVD) of superficial capillary plexus (SCP) and deep capillary plexus (DCP) was quantified in four quadrants (superior, inferior, nasal and temporal).

**Result:**

The mean pVD in SCP and DCP decreased at 6- and 12-months follow-up. In sectoral analysis, superior, inferior, and temporal quadrants pVD in SCP and DCP were significantly reduced at 1 year after vitrectomy (all *p* < 0.05). Meanwhile, inferior quadrants pVD in SCP and DCP showed the earliest significant reduction at 6-months (*p* = 0.022 and 0.048, respectively). A reduction of post-operative mean pVD in DCP was significantly greater in patients with diabetic retinopathy (*p* = 0.043).

**Conclusion:**

Peripapillary capillary density significantly decreased after surgical removal of ERM. Vitrectomy with ILM peeling can cause peripapillary microvascular damage starting in inferior sector.

## Introduction

Idiopathic epiretinal membrane (ERM) is a slowly progressing retinal disease that can cause visual impairment [1]. Pars-plana vitrectomy (PPV) with or without internal limiting membrane (ILM) peeling is required when visual acuity decreases due to membrane progression, and most of the decreased visual acuity can be restored [2, 3]. According to previous studies, gradual visual acuity recovery generally occurs up to three years after surgery, and visual acuity is maintained similarly thereafter [2, 4].

After vitrectomy, clinical findings during post-operative follow-up often include optic nerve head (ONH) pallor or optic nerve atrophy, which sometimes accompanies visual field defects [5, 6]. Post-operative retinal nerve fiber layer changes have been reported in various retinal diseases after PPV [5, 7–9]. Several mechanisms have been hypothesized to explain peripapillary retinal nerve fiber layer (p-RNFL) damage, such as mechanical damage to the inner retinal layers induced by peeling, retinal toxicity caused by the dye, phototoxicity from endo-illumination, and optic disc damage caused by the induction of posterior vitreous detachment. According to a previous report, p-RNFLs tended to decrease in the temporal quadrants of all eyes diagnosed with epiretinal membranes after vitrectomy [10]. Another study reported that p-RNFL showed a tendency to decrease in the inferior quadrants in patients after vitrectomy and ICG-guided ILM peeling, and that p-RNFL thinning is followed by retinal microvascular changes. The post-operative thinning of the inner retinal layer was more prominent in the temporal quadrants than in the nasal quadrants [11]. A previous study in patients with primary open-angle glaucoma (POAG) and disc hemorrhage reported an association between the decrease in p-RNFL thickness and microvascular dropout [12].

Optical coherence tomography angiography (OCTA) is considered a new imaging technique for defining retinal and choroidal microvasculature. Several semi-automatic and automatic programs have been developed to quantify microvascular OCTA parameters [13, 14]. The swept source-OCTA (SS-OCTA) system enables the quantification of microvasculature parameters with a high degree of repeatability and can be used to quantify microvascular changes during follow-up [15]. There have been a few OCTA studies regarding a relationship between PPV and post-operative microvascular changes in parafoveal area [16–18]. More recently, peripapillary microvasculature changes were reported after PPV with retinal detachment repair. They found significant reduction in pVD and RNFL thickness, and significant correlation between two factors which suggest a coexistence of neuronal and microvascular damage [19]. However, only few studies have examined the peripapillary microvascular structural changes and correlated clinical factors after vitrectomy with ILM peeling. Therefore, this retrospective study aimed to investigate peripapillary microvasculature changes in four sectors using SS-OCTA following vitrectomy with ILM peeling for ERM.

## Method

### Study design

The medical records of 33 patients with ERM after surgery were retrospectively reviewed. The patients diagnosed with idiopathic ERM underwent pars plana vitrectomy with ERM and ILM peeling. Patients with cataracts underwent a combined procedure of phacoemulsification and intraocular lens implantation. The subjects visited Kyung Hee university hospital between May 2017 and August 2020. This study was approved by the Institutional Review Board (IRB) of Kyung Hee University Medical Center (2022-06-033) and was conducted in accordance with the tenets of the Declaration of Helsinki. Patients with severe cataracts or corneal opacities were excluded to ensure accuracy of imaging analysis. Patients with severe diabetic retinopathy (DR), vitreous hemorrhage, or diabetic macular edema that precluded good-quality SS-OCTA imaging and examination were excluded. To reduce the signal effect, we excluded images with less than 7/10 signal strength and manually adjusted the threshold value for the peripapillary vessel density (pVD) calculation depending on signal strengths after agreement between the two specialists (KK, KY). Best-corrected visual acuity (BCVA) was converted to the logarithm of the minimum angle of resolution (LogMAR) for statistical analyses. Peripapillary SS-OCTA 6×6 mm^2^ images were measured pre- and post-operatively every 6 months for 1 year. Additionally, patients were divided into two groups according to the presence of DR and analyzed whether there was a statistical difference in pre and post-operative pVD between two groups.

### Surgical procedure

For all 33 patients, 23- or 25-gauge, 3-port PPV was performed with ERM and ILM peeling after staining. In the surgical technique, a 23- or 25-gauge transconjunctival microincision vitrectomy surgery trocars inserted at an angle of 20° to 30°. The infusion cannula was connected to maintain the intraocular pressure. Vitrectomy was performed after making three incisions using the same method. The remaining vitreous was completely removed from 30 eyes (90.9%) using intravitreal triamcinolone acetonide (40 mg/mL; Dong Kwang Pharmaceutical, Seoul, Korea) after posterior vitreous detachment during vitrectomy. ILM rhexis was performed using 23- or 25-gauge microforceps after staining the ILM with 0.25% to 0.5% ICG (Diagnogreen Inj, Daiichi Pharmacy Co, Tokyo, Japan) solution. In all eyes, the ILM was removed from a 2- to 3-disk diameter area centered on the fovea. The IOP was maintained at 20 mmHg during vitrectomy (Alcon CONSTELLATION Vision System). After removing the cannula, leakage was prevented by applying pressure over the scleral incision using a cotton swab. If lamellar macular hole was present, sulfur hexafluoride (SF6) gas tamponade was used at the discretion of the surgeon. Gas tamponade was performed in 12 eyes (36.3%) at the end of surgery.

### SS-OCTA and image processing

All SS-OCTA scans were acquired with 6 x 6 mm^2^ scan centered on the optic nerve head using the PLEX ® Elite 9000 (ZEISS, Dublin, CA) device. This instrument uses a wavelength of 1050 nm and operates at 100,000 A-scans per second. The peripapillary microvasculature was quantified using MATLAB software (R2021a, MathWorks, Inc., Natick, MA, USA) based on an SS-OCTA disc 6×6 mm^2^ image. A custom semiautomatic approach was used to define the region of interest (ROI) in the field of view. The ROI is defined as a 750 μm-wide elliptical ring from the largest diameter of the optic disc [20, 21]. The ONH areas with the largest diameter of the optic disc were manually removed. The large retinal vessels were automatically excluded. Artifacts due to eyeball movement and vitreous opacity were excluded by considering the structural images on SS-OCTA (Fig. 2). We defined a sliding square kernel-based threshold method to define the NPA as a contiguous region without microvasculature. The average signal values within a square kernel of size 17 × 17 (289) pixels centered on each pixel on the SS-OCTA images were used to determine the presence and degree of perfusion of the microvasculature at the pixel location. The threshold for binarizing NPA was based on the method with white and black pixels representing vessels and background or non-perfused regions, respectively [22, 23]. For statistical analysis, the measured pixel-scale NPA was converted into a percentage divided by the total area. The inner boundary of the concentric circle was manually defined as the largest diameter of the optic disc. The definition of vessel density (VD) used the mean binarization threshold [22]. As described, the measured areas, excluding major vessels, were binarized with the mean intensity of the images. The number of pixels over the measured area was counted and converted to pVD. The pVD from the 750 μm-wide ROI was divided into four quadrants (superior, inferior, temporal and nasal) (Fig. 2).

### Statistical analysis

Statistical analyses performed using Statistical Product and Service Solutions (version 23.0; SPSS Inc., Chicago, IL, USA). The repeated measures-analysis of variance (RM-ANOVA) method was used to compare baseline pVD and at each follow-up visit. The post-hoc analysis was performed using the Bonferroni method. Comparisons between the parameters at baseline were performed using Fisher’s exact test and the Mann–Whitney U-test. Wilcoxon’s Rank test was used to evaluate the OCTA parameter differences between DR and no-DR patients. p-values <0.05 were considered statistically significant. In the post-hoc analysis between the two groups, significance was confirmed based on the more stringent criteria of the Bonferroni method.

## Results

**Fig. 1:**
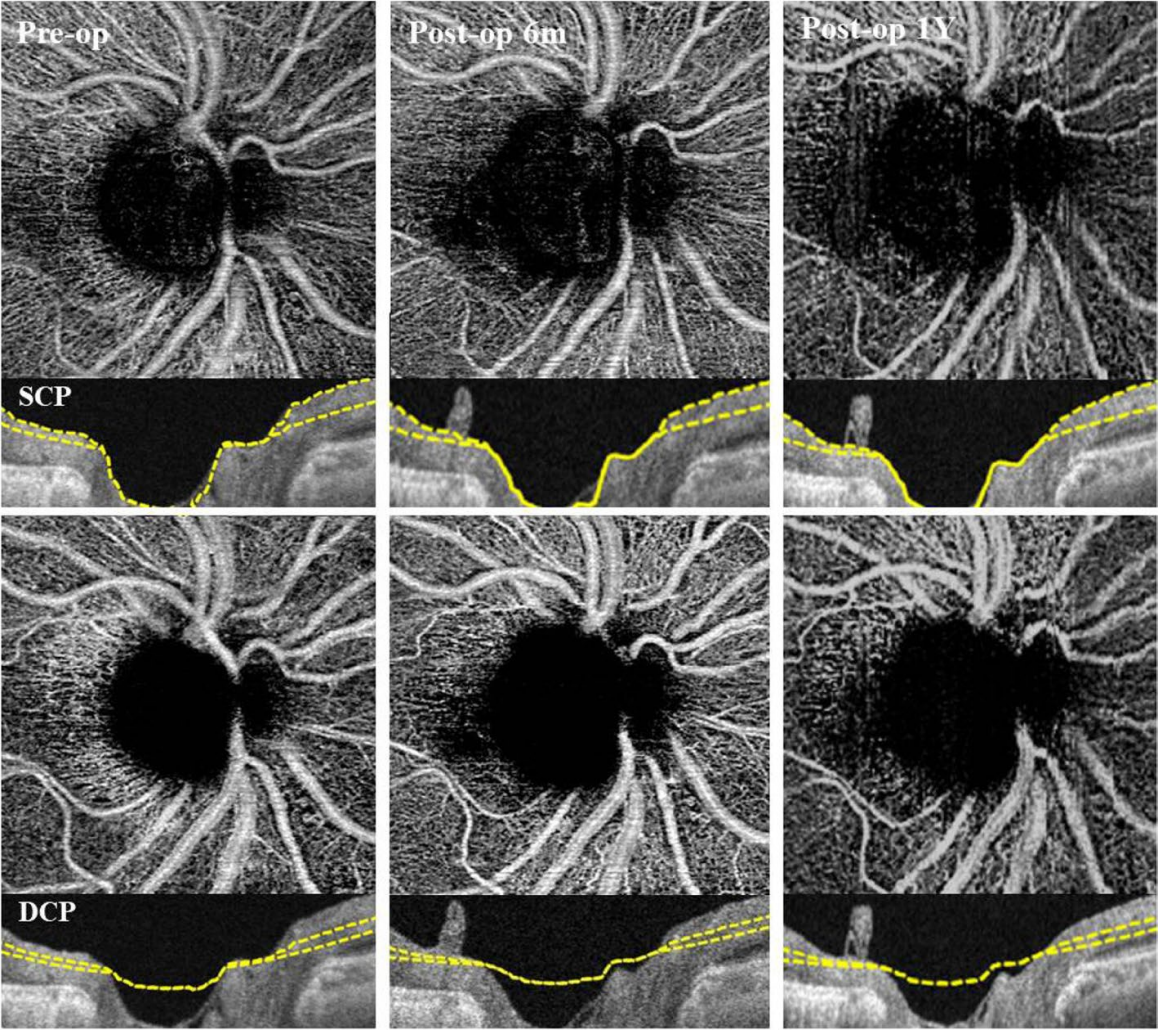
Representative swept-source optical coherence tomography angiography (SS-OCTA) images of patients with ERM after PPV and ILM peeling. Pre- and post-operative SS-OCTA images in superficial capillary plexus and deep capillary plexus. The Inferior and temporal quadrant peripapillary microvasculature prominently decreased during the follow-up

**Fig. 2:**
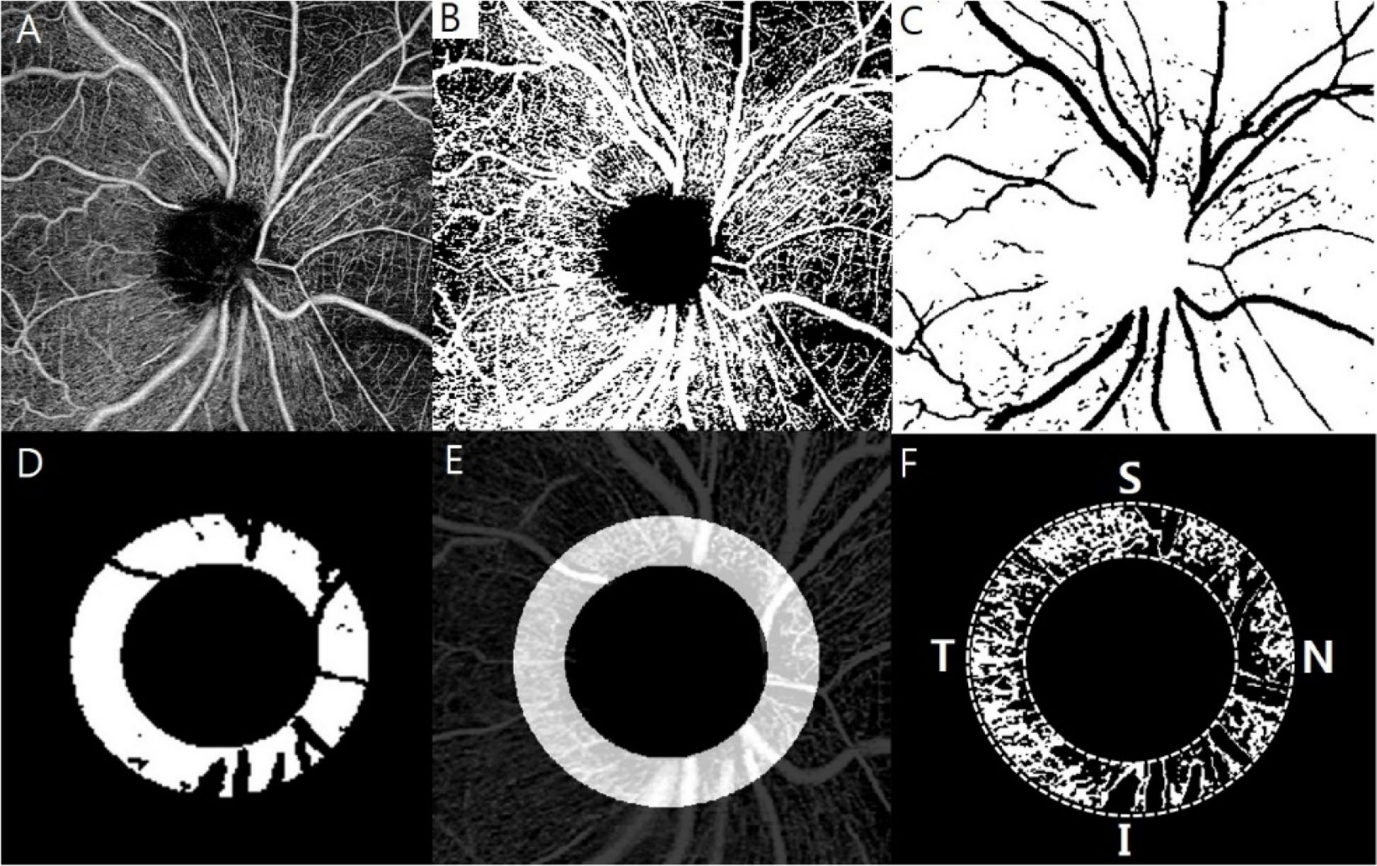
Representative peripapillary swept-source optical coherence tomography angiography (SS-OCTA) images and peripapillary vessel density of the iamges. **A** The 6 × 6 mm^2^ peripapillary SS-OCTA images. **B** The binarizing images based on the method with white and black pixels representing vessels and background or non-perfused area (NPA). **C** Automatically excluded large retinal vessel. **D** Defining region of interest (ROI) on the retinal slab covering 6 × 6 mm^2^ peripapillary SS-OCTA image excluding the optic disc and large retinal vessels. **E** The ROI defined as a 750 μm-wide elliptical ring from the largest diameter of the optic disc. **F** The measured pixel-scale NPA was converted into a percentage divided by the total area, and automatically calculated peripapillary vessel density in 4 quadrant (Superior, Temporal, Inferior and Nasal)

A total of 33 eyes of 33 patients with ERM after PPV and ILM peeling were analyzed. The demographic and baseline characteristics of the participants are presented in Table 1. Representative pre- and post-operative SS-OCTA images are shown in Fig. 1, and images of the pVD analysis using MATLAB are shown in Fig. 2. The four quadrants of pVD in the superficial and deep capillary plexus (SCP and DCP) were calculated using MATLAB. Correlations between four-quadrant pVD and baseline clinical characteristics, including age, BCVA, pre-and post-operative intraocular pressure, were not statistically significant (all *p* < 0.05).

**Table 1:** Demographics and Comparison of clinical characteristics of eyes with DR and no-DR

Demographics and Clinical Features			
Number of eyes	33		
Sex, male/female	8/25		
Age, years	67.75 ± 6.92		
Diabetes, n (%)	13 (39.4%)		
Hypertension, n (%)	21 (63.6%)		
Glaucoma History, n (%)	4 (12.1%)		
IGC-guided ILM peeling, n (%)	33 (100%)		
Intravitreal Gas (SF_6_) injection	13 (39.4%)		
Preoperative Lens Status (n=Phakic) (%)	24/33 (72.7%)		
BCVA (Baseline), logMAR	0.42 ± 0.31		
IOP (Baseline), mmHg	14.97 ± 2.92		
IOP (Post-op, 6Mo), mmHg	13.53 ± 3.94		
IOP (Post-op, 1Y), mmHg	14.15 ± 3.84		
Axial length, mm	23.85 ± 1.12		
	DR	no-DR	*p*
Number of eyes	13	20	
Sex, male/female	4/11	4/16	.487
Age, years	68.92 ± 6.8	67.00 ± 7.1	.573
Glaucoma History, n (%)	1 (7.6%)	3 (15.0%)	.976
Intravitreal Gas (SF_6_) injection	5 (38.5%)	8 (40.0%)	.931
Preoperative Lens Status (n=Phakic) (%)	10/13 (76.9%)	14/20 (70%)	.667
BCVA (Baseline), logMAR	0.47 ± 0.27	0.39 ± 0.30	.221
IOP (Baseline), mmHg	14.69 ± 3.4	15.15 ± 2.64	.957

*ICG *Indocyanin green,* ILM *Internal limiting membrane,* BCVA *Best corrected visual acuity,* logMAR *Logarithm of the minimum angle of resolution,* IOP *Intraocular pressure

**Fig. 3:**
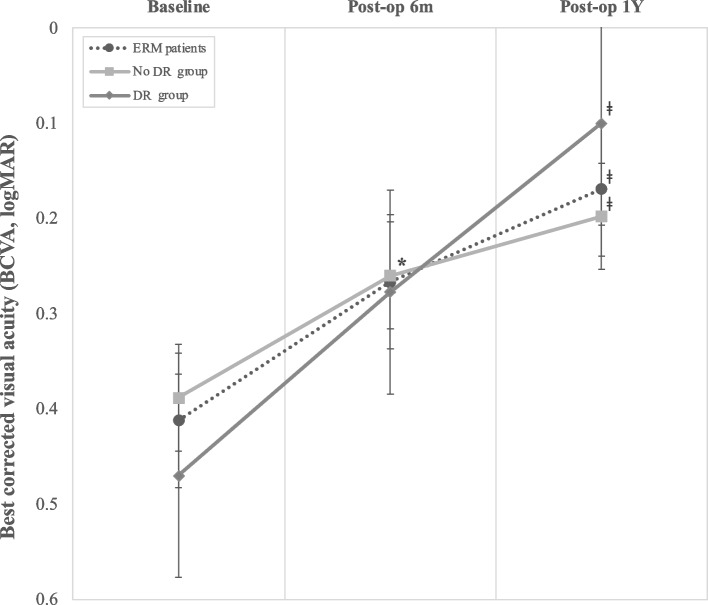
Best corrected visual acuity (BCVA) at baseline, six months and one year postoperatively in diabetic retinopathy (DR) group and no DR group. *: p < 0.05, Compare pre-op to post-op 6months, ǂ: p < 0.05, Compare pre-op to post-op 1 year

BCVA showed significant improvement up to one year after vitrectomy (*p* = 0.001). In addition, mean BCVA was significantly improved following both groups, regardless of the presence of DR (DR and no-DR groups) (Fig. 3). There was no significant difference in BCVA between the two groups during the 1-year follow-up period

### Superficial and deep capillary plexus pVD

**Fig. 4:**
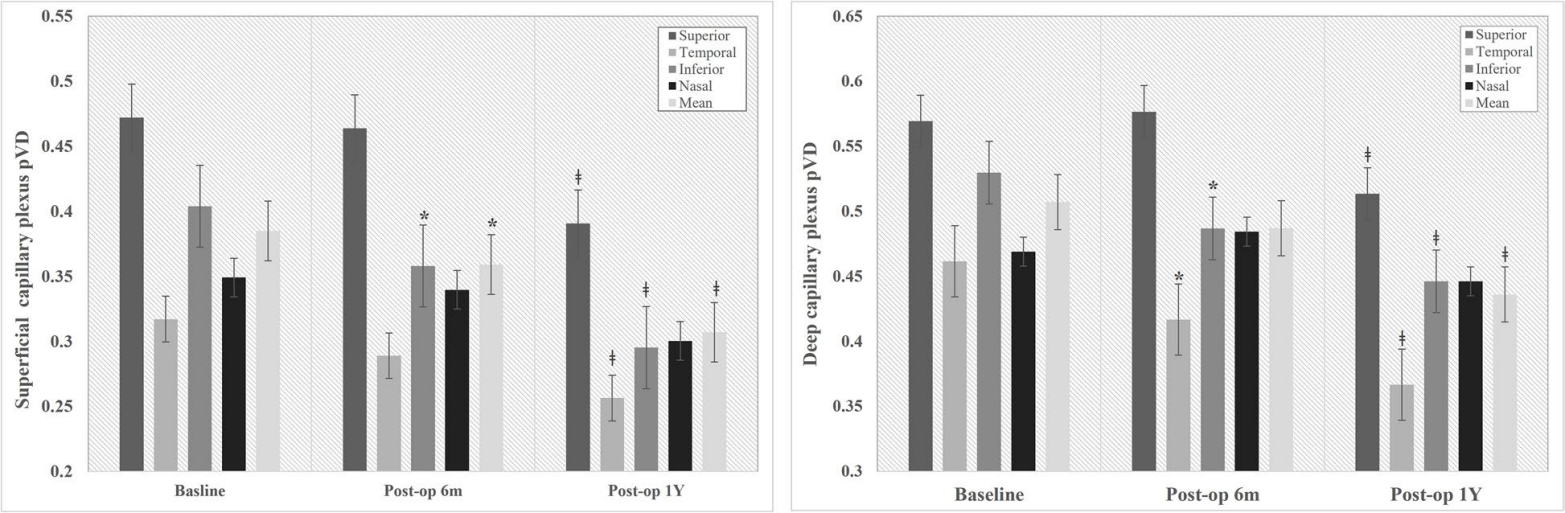
Comparison of peripapillary vessel density (pVD) at baseline versus post-operative 6 months and 1 year in superficial capillary plexus and deep capillary plexus *: *p* < 0.05*, Compare pre-op to post-op 6months,* ǂ: *p* < 0.05*, Compare pre-op to post-op 1 year*

The mean pVD in SCP significantly decreased at both 6- and 12-months follow-up. In sectoral analysis, inferior quadrant pVD showed significant reduction at 6- and 12-months follow-up (*p* < 0.05, respectively). Superior and temporal quadrant pVD in SCP significantly decreased at 12-months after vitrectomy (*p* = 0.004 and 0.001, respectively). A similar reduction trend in mean pVD was observed in DCP. The temporal and inferior quadrant pVD in DCP significantly decreased at both 6- and 12- months follow-up, while superior quadrant pVD decreased at 12 months after vitrectomy (*p* < 0.05, respectively) (Fig. 4).

### Subgroup analysis of pVD by DR

Of the 33 patients, 13 (39.4%) presented DR and 20 (60.6%) had no-DR. In patients with DR, the temporal and inferior quadrant pVD in SCP significantly decreased at post-op 1-year (*p* = 0.001 and 0.012, respectively). The patients without DR had a prominent pVD decrease in the inferior quadrant in SCP and in the temporal and inferior quadrants in DCP (*p* < 0.05, respectively). The difference of pVD in SCP and DCP between DR and no-DR groups were described in Table 2. Post-operative mean pVD in DCP was significantly lower in patients with DR than that of no-DR at post-op 6- and 12-months (*p* = 0.015 and 0.043, respectively).

**Table 2:** The difference of pVD between no-DR group and DR group on SCP and DCP

		SCP pVD					DCP pVD				
*The difference of pVD* *(no-DR* *vs. DR)*		Sup	Temp	Inf	Nasal	Mean	Sup	Temp	Inf	Nasal	Mean
	Baseline	0.023	-0.008	-0.016	-0.003	-0.001	0.023	-0.013	0.012	0.006	0.008
	6 months	0.091	0.059	-0.001	0.080	0.060*	0.130*	0.075	0.056	0.086	0.086*
	1 year	0.042	0.045	0.043	0.051	0.046	0.080	0.041	0.034	0.066	0.055*

(The difference of pVD) = (The mean pVD of no-DR patients) - (The mean pVD of DR patients), pVD Peripapillary vessel density, SCP Superficial capillary plexus, DCP Deep capillary plexus, Sup Superior, Temp Temporal, Inf Inferior

^*^p < 0.05, statistically significant comparing no-DR and DR group

## Discussion

This study investigated serial changes in the peripapillary microvasculature in the ERM after PPV with ILM peeling. The mean BCVA significantly improved after vitrectomy. The post-operative mean pVD in SCP and DCP progressively decreased over 1 year. A post-operative pVD decrease was evident in the inferior, temporal, and superior quadrants.

Our results showed that post-operative BCVA in patients with ERM demonstrated progressive improvement and stably maintained during 1 year follow-up. It was previously reported that poorer pre-operative BCVA group improved with a larger proportional magnitude within 1 year [4]. We also found a significant BCVA improvement in both DR and no-DR patients, and the difference was not statistically significant. The pre-operative BCVA was better in patients without DR, whereas the DR group showed a better BCVA at 1 year. Other pre-operative clinical factors, including age, lens status (phakic or pseudophakic), and glaucoma history, were not significantly different. Previous studies have reported a correlation between pre- and post-operative BCVA [24, 25], while others have not [4, 26]. In this study, we could not find a correlation between pre- and post-operative BCVA. This discrepancy may be due to other clinical factors affecting BCVA such as pre-operative cataract grade and selection bias related to DR severity. Therefore, further associations between visual recovery and these factors need to be identified.

Our study demonstrated significant changes of the peripapillary microvasculature in SCP and DCP after PPV. Previous studies have found that the p-RNFL tended to decrease in the inferior and temporal areas after vitrectomy for ERM [7, 11]. This selective decrease in the inferior and temporal p-RNFL thickness after vitrectomy could be associated with inner retinal damage caused by ILM peeling [27, 28]. Mechanical damage to superficial retinal vessels may cause transient ischemic changes in the inner retinal layers. In other reports, RNFL thinning seemed to correlate with vascular changes in patients with POAG or DR [29–31]. Dysfunction of the microvasculature or abnormal blood flow in the retinal capillary plexus may lead to adverse effects on retinal nerve functions, including RNFL or ganglion cells [28]. Previous studies demonstrated that the pVD of inferior and temporal sector have the strongest association with the corresponding visual sensitivity loss and circumpapillary RNFL (cpRNFL) thickness in POAG eyes [32, 33]. The localized inferior or inferotemporal cpRNFL thinning is the earliest glaucomatous sign in POAG [34, 35]. It has been related to the least supporting connective tissue and the larger single pores of lamina cribrosa in the inferior temporal region. Less structural support and anatomical features of inferior and temporal region contributes to more susceptible to ischemic changes. Holló et al. suggested that measuring the inferior and temporal peripapillary microvascular density could identify glaucomatous damage earlier than measuring the corresponding RNFL thickness [36]. According to the data from this study, the inferior and temporal quadrant pVD in SCP and DCP were prominently decreased at the end of the follow-up. Chronic inflammation and secondary ischemic changes of retina can be a major cause of postoperative decrease in pVD. Since inferior temp disc sector is structurally weaker than other area, inferior and temporal peripapillary capillary can be more vulnerable to ischemic damage without direct surgical trauma. This result corroborated earlier findings of decreased inferior and temporal p-RNFL after surgery [11].

We investigated the difference of pre- and post-op pVD between in patients with DR and no-DR. ERM patients with DR had significantly lower mean pVD in DCP after 1 year. Several studies reported the association between reduction of peripapillary microvasculature and DR. Vujosevic et al. evaluated the peripapillary retinal microvasculature in the radial plexus and superficial macular layer among healthy controls, diabetes without DR, and mild NPDR. They demonstrated a significant decrease in the peripapillary area VD, which was followed by the macular region in the DR group versus controls [31]. Peripapillary microvasculature alterations in patients with DR could be considered early preclinical signs of diabetic microvascular disease [31, 37]. Greig et al. found that pVD reduction in the superior, inferior, and temporal areas was significantly associated with DR progression [38]. Reactive gliosis is one of the early pathological characteristics of DR. Activated glial cells induce chronic inflammatory response, which cause a loss of capillaries and progressive retinal ischemia during DR [39]. Meanwhile, under injury or ischemic stress by ERM surgery, microglial cells also get activated and proliferate, releasing pro-inflammatory cytokines to counter the damage. Therefore, we could hypothesize that postoperative decrease of pVD might be more significant in DR patients due to retinal ischemic cascade.

A previous study reported that the fractal dimension used for quantitative analyses (such as VD) of DM without DR significantly decreased in the macular area compared to healthy controls. This reduction was evident in the DCP. This may be due to the greater microvasculature density in DCP than in SCP, in which vascular constriction of the DCP in the macular region may be an earlier compensating mechanism than in SCP for decreased blood flow, resulting in hypoxia and ischemia in patients with diabetes [36]. We documented prominent peripapillary microvasculature reduction in DCP in DR patients. Although changes of VD from the natural course of DR was not considered in this study, we initially excluded the eyes with severe stage of DR and collected data during the first year after surgery to minimize possible effect. Thus, monitoring of pVD changes after PPV may be recommended after PPV in ERM patients with DR.

Current study has several limitations, such as the number of enrolled patients was limited to 33, with a relatively small sample size. Second, projection artifacts on SS-OCTA images caused by vitreous opacity or cataracts may mask the pre- and post-operative microvasculature, and the shadowed area may affect vessel density. To reduce false positives, we excluded patients with severe cataracts or severe DR. However, still shadowed areas might not have been completely excluded, and selection bias may have affected the study results. Third, the low overall signal strength of the images can affect the pVD value. Fourth, the evaluation of anatomical or functional changes, such as RNFL thickness and visual field test, were not included in this study. Further studies with a larger prospective design will provide a better understanding of microvascular changes and functional and anatomical results. Lastly, proper segmentation is crucial for layer-by-layer (SCP and DCP) analysis of SS-OCTA images. We managed the quality of segmentation through confirmation of two specialists (KK, KY). Nevertheless, segmentation errors may exist and this limitation expected to overcome through the development of algorithms capable of more accurate segmentation or layer classification through higher resolution in future studies.

In conclusion, the peripapillary microvasculature significantly decreased following ERM surgery, in particular, inferior sector pVD prominently decreased after 6 months. Vitrectomy with ILM peeling can cause indirect damage to peripapillary capillary plexus. Further studies are needed to investigate causal relationship between microvascular and neuronal damage and possible role of pVD in predicting RNFL thinning after ERM surgery.

## Data Availability

The datasets used and/or analysed during the current study are available from the corresponding author on reasonable request.
